# Enhancing growth-relevant metabolic pathways of *Arthrospira platensis* (CYA-1) with gamma irradiation from ^60^Co†

**DOI:** 10.1039/c8ra01626g

**Published:** 2018-05-08

**Authors:** Jun Cheng, Hongxiang Lu, Ke Li, Yanxia Zhu, Junhu Zhou

**Affiliations:** State Key Laboratory of Clean Energy Utilization, Zhejiang University Hangzhou 310027 China

## Abstract

The biomass yield of *Arthrospira* mutant ZJU9000 was 176% higher than that of wild type on day 4, and the results of transcriptome sequencing showed that processes related to cell growth were synergistically enhanced in this mutant. The amount of energy for biomass accumulation increased because the efficiency of the photoreaction was enhanced by the elevated levels of chlorophyll *a* and carotene. The increased biosynthesis rates of ribose phosphate, nucleotides and multiple vitamins increased the production of genetic materials for cell proliferation. Furthermore, the carbon concentration mechanism in mutant ZJU9000 was enhanced, indicating the increased utilization efficiency of CO_2_ at low concentration (0.04 vol% in air). The enhancement of these growth-relevant metabolic pathways contributed to the robust growth of *Arthrospira* mutant ZJU9000.

## Abstract 2

Growth-relevant metabolic pathways of *Arthrospira* enhanced after gamma irradiation.

## Graphical abstract

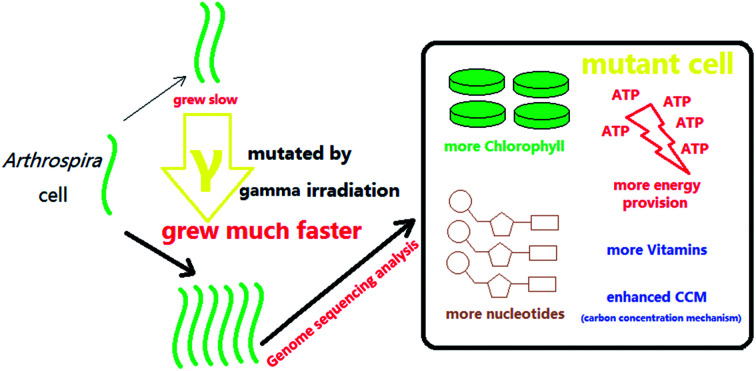

## 1. Introduction

Phytoplanktons, including cyanobacteria as a major group, are responsible for approximately 50% of photosynthesis worldwide, thereby dominating the global conversion of CO_2_.^1^*Arthrospira* (*Spirulina*) is one of the fastest growing cyanobacteria. The taxonomic relationship between *Arthrospira* and *Spirulina* has been unclear for a long time.^2^*Spirulina* was later found to be a prokaryotic cyanobacterium belonging to the genus *Arthrospira*,^3^ thus the name *Arthrospira* is adopted in this study (we used the term “*Spirulina*” in our previous studies). Improving *Arthrospira's* growth rate increases the amount of CO_2_ it can assimilate and alleviates the greenhouse effect. Moreover, its biomass can be used as food and feed supplements.^4,5^ Performing genetic analysis on *Arthrospira* can help us further understand the mechanisms underlying different growth phenotypes between strains. Two resources allowed us to investigate different *Arthrospira* phenotypes at the genetic level. Firstly, the genome sequence of *Arthrospira* sp. PCC9438 became available,^6^ and thus effective gene identification through high-throughput sequencing technology has become feasible.^7^ Secondly, the Kyoto Encyclopedia of Genes and Genomes (KEGG) resource (http://www.genome.jp/kegg/) provided a reference knowledge database for linking genomes to biological systems and the wiring diagrams of interaction networks and reaction networks (KEGG pathway). The continuous increase in the amount of genomic and molecular information has enabled researchers to understand higher order biological systems, such as cells and organisms, and their interactions with the environment.^8^

The gene expression levels and metabolism of *Arthrospira* under various conditions were investigated in some studies, such as that of Badri *et al.*, who irradiated *Arthrospira* sp. PCC8005 by ^60^Co gamma-ray irradiation at various dosages and analysed the differences in gene expression levels between wild type and irradiated strains at different healing times (0, 2 and 5 h). They found that the *Arthrospira* pathways, including the tricarboxylic acid (TCA) cycle and pentose phosphate pathway, changed significantly in response to abrupt and intense radiation.^9^ Panyakampol *et al.* showed that several heat shock proteins of *Arthrospira platensis* C1 exhibited rapidly increasing transcription levels in response to high temperature.^10^ Wang *et al.* analysed differentially expressed genes during protein coding and relevant metabolic pathways in *Arthrospira* under various temperatures and found that differentially expressed positive proteins were mainly involved in post-translational modification and energy metabolism.^11^ Esen and Ozturk Urek reported that the iron and ammonium nitrate concentrations for the optimum growth, pigment and metabolite levels and enzyme activities of *Arthrospira* cells were 50 × 10^−6^ mol L^−1^ and 10 × 10^−3^ mol L^−1^, respectively.^12^ However, only the gene expression levels of *Arthrospira* cells in response to various cultivate conditions were considered in these studies, and no mutant with hereditarily stable phenotypes were obtained. Abomohra *et al.* irradiated *Arthrospira platensis* with gamma rays and found that 2 and 2.5 kGy dosages inhibited its biomass production. They also investigated changes in the cellular components of these species under various radiation dosages. Their results indicated that carotenoid productivity greatly increased significantly after irradiation, whereas the contents of other major components, such as chlorophyll *a* and protein, decreased.^13^ Although Abomohra's study comprehensively investigated the influence of gamma radiation on *Arthrospira* cells, all the analyses were performed at the macro level and lacked the perspective view at the genetic-level.

ZJU9000 is a hereditarily stable *Arthrospira* mutant culture obtained after 9 kGy gamma irradiation exhibiting a robust growth in respect to the wild type.^14^ In the present study, the difference in gene expression levels between the wild type and mutant ZJU9000 were analysed. The purpose was to investigate the underlying cause of the mutant's robust growth. Transcriptome sequencing showed that many key genes of the mutant ZJU9000 cells were expressed at much higher levels than those of the wild type, such as the genes related to photosynthetic pigments synthesis, nucleotides synthesis, glycolysis and TCA cycle. Expressions of these genes provided the materials and energy basis for cell proliferation. Moreover, mutant ZJU9000 captured more CO_2_ at low concentrations than wild type *Arthrospira* owing to its improved carbon concentration capability. These results elucidated the robust growth of *Arthrospira* ZJU9000.

## 2. Materials and methods

### 2.1. *Arthrospira* strains and preparation of samples

Wild type *Arthrospira* (*Spirulina*) *platensis* (CYA-1) was obtained from Marine Biological Culture Collection Center, Institute of Oceanology, Chinese Academy of Sciences. *Arthrospira* mutant culture ZJU9000 was induced by gamma rays irradiation from ^60^Co, detailed gamma radiation-induced mutation method was demonstrated in our previous work.^14^ Wild type *Arthrospira* and mutant ZJU9000 were cultivated under continuous light source of 8000 Lux supplied at the surface of bioreactors using four cool white lights (Philips, TLD 36 W, 927982286574) and two warm lights (Philips, TLD 36 W, 927982283074). Microalgae cultures in the bioreactors were bubbled with air at a flow rate of 30 ml minute^−1^ (CO_2_ concentration = 0.038 vol%). Algae species were cultivated at 25 °C in column bioreactors (160 × 10^−3^ m height, 56 × 10^−3^ m inner diameter and 60 × 10^−3^ m outer diameter) with 300 ml working column in an artificial greenhouse. Specific growth rate *μ* of microalgae was calculated as follows:
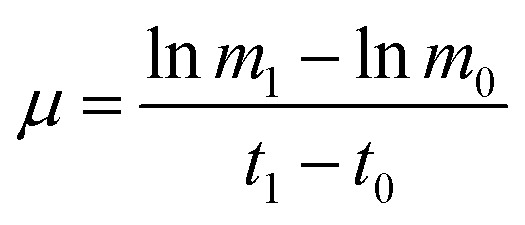
where *m*_1_ was the dry weight at time *t*_1_ and *m*_0_ was the dry weight at time *t*_0_, *t*_1_ is the day after *t*_0_.

On day 4, cultures of wild type *Arthrospira* and ZJU9000 were filtered, quick-frozen in liquid nitrogen, collected and stored at −80 °C before further analysis.

### 2.2. Pigments and photosynthetic efficiency measurement

Pigments and photosynthetic efficiency of wild type *Arthrospira* and mutant ZJU9000 were measured on the 4^th^ day.

Chlorophyll *a* and carotenoid content were measured by chemical method following Pruvost's protocol.^15^ 1 ml microalgae culture was filtered and the pellet was extracted with 3 ml methanol (≥99.9%) for 30 min in the dark at 45 °C. Supernatant was filtered and absorption spectra were measured by spectrophotometer UV-3200B (Mapada instruments, Shanghai, China) at wavelength 480, 652 and 665 × 10^−9^ m (*A*_480_, *A*_652_ and *A*_665_). Chlorophyll *a* and carotenoid concentrations were calculated according to the equations of Lima^16^ (chlorophyll *a*) and Strickland and Parsons^17^ (carotenoid) as follows:chlorophyll *a* (μg ml^−1^) = 12.6 × *A*_665_carotenoid (μg ml^−1^) = 4 × *A*_480_

Photosynthetic efficiency was reflected by *F*_v_/*F*_m_ value (FMS-2, Hansatech, UK) of microalgae culture. 2 ml microalgae sample was added into the test tube followed by a 10 min dark adaption. Then the microalgae sample was exposed to a saturation light pulse and *F*_0_ and *F*_m_ were measured. *F*_v_/*F*_m_ value was calculated as follows:*F*_v_/*F*_m_ = (*F*_m_ − *F*_0_)/*F*_m_where *F*_0_ was the minimum fluorescence (basic fluorescence) and *F*_m_ was the maximum fluorescence.

### 2.3. RNA extraction, library preparation and bioinformatics analysis

RNA was extracted from collected samples (in Section 2.1) of both wild type and mutant ZJU9000, followed by the library preparation and bioinformatics analysis. We followed manufacturer's protocol mirVana miRNA kit (Thermofisher) for RNA extraction, Truseq stranded mRNA LT sample prep kit (Illumina) for sequencing library preparation, and Bioanalyzer's High Sensitivity DNA kit (Agilent) for quality control of libraries. Specific procedures were described in the ESI.† The transcriptomes of wild type and mutant ZJU9000 were subsequently compared to each other.

### 2.4. Figures of metabolic pathway and colours in figures

Kyoto Encyclopedia of Genes and Genomes (KEGG) mapping was used to characterise the metabolic pathways (http://www.genome.jp/kegg/pathway.html). The uncoloured pathway map was the original version which was manually drawn by the in-house software, KegSketch. In this study, organism-specific pathway was chosen and “*Arthrospira platensis* NIES-39” was selected in “reference pathway”. Specific genes and enzymes which belonged to *Arthrospira* automatically showed up as green on the map. Among these specific genes and enzymes, up-regulated and down-regulated differentially expressed genes (DEGs) were coloured with red and blue, respectively.

## 3. Results and discussion

### 3.1. Difference in growth phenotype and general analysis of DEGs between wild type *Arthrospira* and ZJU9000

**Fig. 1:**
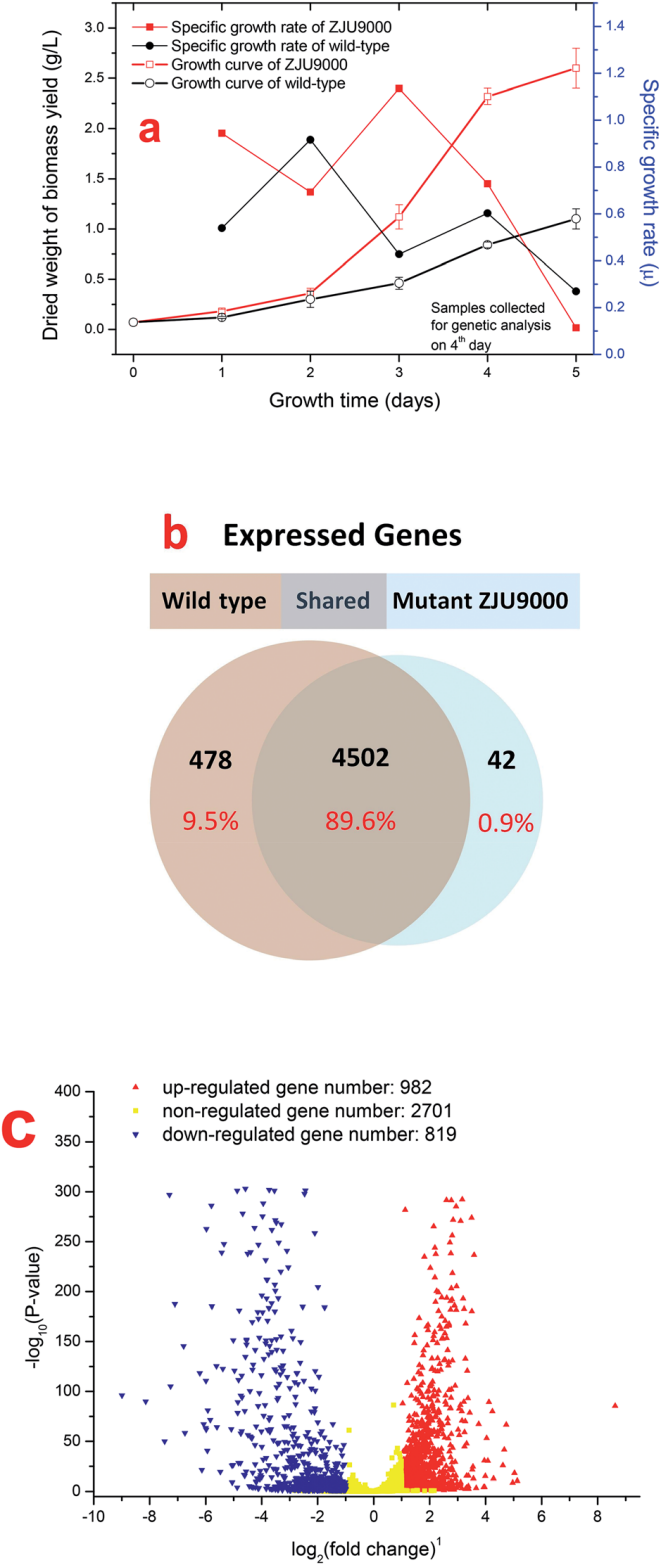
Growth phenotype and general analysis of DEGs in wild type *Arthrospira* and mutant ZJU9000. (a) Growth curve and specific growth rate of wild type *Arthrospira* and mutant ZJU9000. (b) Venn diagram of shared and unique expressed genes in wild type *Arthrospira* and mutant ZJU9000. (c) Scatter diagram of DEGs: *X*-axis shows the logarithm of FPKM ratio between wild type and mutant, *Y*-axis shows the significance of DEGs (the value higher than 1.3 means *p* < 0.05). Red represents the 982 up-regulated genes, blue represents the 819 down-regulated genes and yellow represents the 2701 non-DEGs. Note 1: 
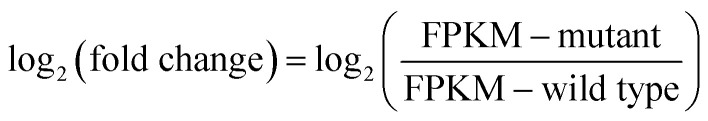
, FPKM: fragments per kilobase of exon per million fragments mapped.

The growth curves and specific growth rates of wild type *Arthrospira* and mutant ZJU9000 were shown in Fig. 1a. The biomass yield and highest growth rate of the mutant ZJU9000 were both higher than those of the wild type. The peak specific growth rate of mutant ZJU9000 (1.13) was 23% higher than that of wild type (0.92). The growth curves of both strains in this study were different from our previous work^14^ because the inoculation stage of microalgae might have been different in diverse batches of cultivation. However, the biomass yield of ZJU9000 on day 4 was 176% higher than wild type, and the boosted biomass yield in ZJU9000 was consistent with the results in our previous work.

High-throughput sequencing was used for the systematic analyses of the gene expressions of wild type *Arthrospira* and mutant ZJU9000 cells. The purpose was to identify the differences in metabolic pathways, especially those that led to the mutant's increased biomass yield. A total of 16 923 580 and 12 718 076 high-quality clean reads were identified from the wild type and mutant *Arthrospira*, respectively.

The correlation coefficient of gene expression level between the two samples represented their similarity. If the coefficient was closer to 1, then the similarity between them was higher.^18^ Samples belonging to biological replicates should have a coefficient higher than 0.92. However, the correlation coefficient between the wild type *Arthrospira* and ZJU9000 was only 0.34, indicating the dramatic variation between the two strains.

As shown in Fig. 1b, 4502 genes were expressed both before and after mutation. Among these shared genes, 1801 genes were differentially expressed, of which 982 were up-regulated and 819 were down-regulated (Fig. 1c). Particularly, the unique expressed genes in one strain (unexpressed genes in the other strain) were also counted as DEGs and were included in subsequent analyses.

**Fig. 2:**
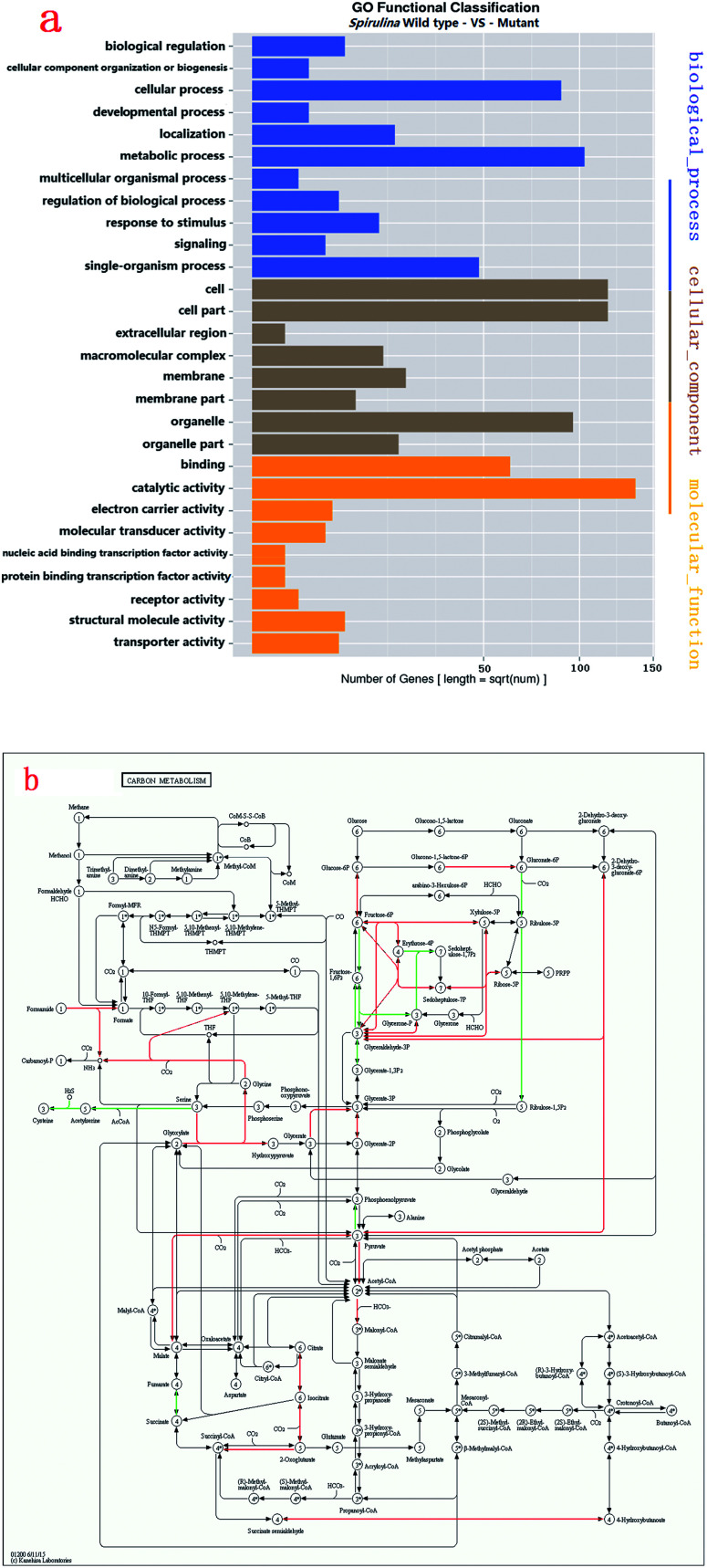
DEGs in *Arthrospira* wild type and mutant ZJU9000. (a) Gene ontology (GO) function significant enrichment analysis showed the distribution of DEGs in biological process, cellular component and molecular function. (b) Many genes involved in Calvin cycle, TCA cycle, glycolysis, pentose phosphate pathway and C4-dicarboxylic acid cycle were highly overexpressed in carbon metabolism. (a) Distribution of GO function enrichment between wild type *Arthrospira* and mutant. (b) DEGs in carbon metabolism (red lines represent up-regulated pathways, green lines represent down-regulated pathways).

GO enrichment analysis was performed on 1801 DEGs on the basis of the GO database to further explore the biological function of DEGs derived by gamma radiation from ^60^Co. Catalytic activity had the highest number of DEGs, followed by cell/cell part and metabolic process (Fig. 2a). The results of the GO enrichment analysis showed that catalytic activities, cell part and metabolic process in ZJU9000 significantly changed, thereby affecting the growth phenotype and CO_2_ biofixation pathways of the mutant.

The KEGG analysis results on carbon metabolism (Fig. 2b) showed the overexpression of TCA cycle, glycolysis, pentose phosphate pathway, Calvin cycle and C4-dicarboxylic acid cycle genes. These pathways, such as energy provision *in vivo*, synthesis of genetic materials and carbon fixation, were associated with the cell growth and proliferation of *Arthrospira*. The up-regulation of these genes proved the enhancement of these growth relevant pathways.

### 3.2. Enhancing photosynthetic process by improving pigment synthesis in ZJU9000

Natural pigments, such as chlorophyll *a* and carotene, had important roles in the photosynthetic and pigmentation metabolism of microalgae.^19,20^ The maximum absorption wavelengths of chlorophyll *a* and carotene were 420–663 and 448 × 10^−9^ m, respectively. The different maximum absorption wavelengths of these pigments jointly contributed to the comprehensive utilization of light energy by cells.^21–23^

**Fig. 3:**
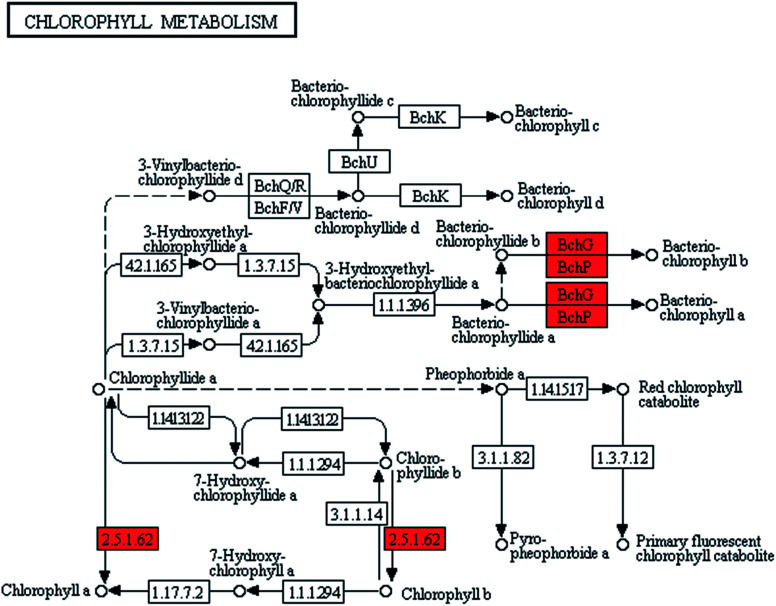
Transcript expression changes involved in chlorophyll metabolism of ZJU9000 (Coloured boxes are *Arthrospira*-specific enzymes based on references, red boxes represent up-regulated genes).

Pigments synthesis was improved in ZJU9000 because the relevant genes were highly overexpressed (Fig. 3). The *bchG* and *bchP* genes, which jointly encoded chlorophyll synthase, the most important enzyme in chlorophyll biosynthesis (eqn (1)), had considerably higher expression levels than those in the wild type (Table 1).

1 Chlorophyllide *a* + phytyl diphosphate → chlorophyll *a* + diphosphate

**Table 1:** Annotation and regulation of differentially expressed key genes in *Arthrospira* wild type and mutant

EC No.^a^	Relevant gene name	KO^b^_definition	Pathway involved	FPKM^c^	
				Wild type	Mutant ZJU9000
2.5.1.62	*bchG*	Chlorophyll synthase	Chlorophyll metabolism	132.1	323.28
1.3.1.83	*bchP*	Geranylgeranyl reductase	Chlorophyll metabolism	93.78	642.44
5.2.1.12	*z-iso*	Zeta-carotene isomerase	Carotenoid biosynthesis	108.31	631.62
5.2.1.13	*crtiso*	Prolycopene isomerase	Carotenoid biosynthesis	115.25	720.09
1.3.99.30	*al1*	Phytoene desaturase (3,4-didehydrolycopene-forming)	Carotenoid biosynthesis	120.14	584.83
5.3.1.9	*gpi*	Glucose-6-phosphate isomerase	Pentose phosphate; glycolysis	122.58	407.67
2.2.1.1	*tkt*	Transketolase	Pentose phosphate	123.84	273.19
2.2.1.2	*tal*	Transaldolase	Pentose phosphate	118.65	527.4
2.7.1.15	*rbks*	Ribokinase	Pentose phosphate	148.99	582.56
2.7.4.8	*gmk*	Guanylate kinase	Purine metabolism	139.02	573.63
3.6.1.19	*itpa*	Inosine triphosphate pyrophosphatase	Purine metabolism; pyrimidine metabolism	91.76	464.34
2.4.2.10, 4.1.1.23	*umps*	Uridine monophosphate synthetase	Pyrimidine metabolism	94.04	245.88
2.4.1.13		Sucrose synthase	Sucrose biosynthesis	111.3	241.82
3.2.1.21	*bglX*	Beta-glucosidase	Sucrose biosynthesis	144.08	343.15
2.7.1.4	*scrK*	Fructokinase	Fructose biosynthesis	100.31	237.94
1.8.1.4	*dld*	Dihydrolipoamide dehydrogenase	Glycolysis; TCA cycle	106.38	335.92
2.3.1.12	*dlat*	Dihydrolipoamide acetyltransferase	Glycolysis; TCA cycle	103.12	219.47
4.2.1.3	*aco*	Aconitate hydratase	TCA cycle	182	476.36
5.5.1.24	*vte1*	Tocopherol cyclase	Vitamin E biosynthesis	122.67	327.11
2.1.1.295	*vte3*	MPBQ/MSBQ methyltransferase	Vitamin E biosynthesis	99.16	216.1
2.1.1.95	*vte4*	Tocopherol *O*-methyltransferase	Vitamin E biosynthesis	60.58	170.41
3.5.4.26, 1.1.1.193	*ribD*	Diaminohydroxyphosphoribosylaminopyrimidine deaminase	Vitamin B_2_ biosynthesis	90.63	380.67
2.3.1.47	*bioF*	8-Amino-7-oxononanoate synthase	Biotin biosynthesis	174.37	2146.57
1.1.1.100	*fabG*	3-Oxoacyl-[acyl-carrier protein] reductase	Biotin biosynthesis	175.55	734.8
2.1.1.163	*ubiE*	Demethylmenaquinone methyltransferase	Vitamin K1, K2 biosynthesis	95.06	196.79
1.1.1.39, 1.1.1.40	*maeB*	Malate dehydrogenase	Carbon fixation	88.19	193.95

a Enzyme commission number.

b Kyoto Encyclopedia of Genes and Genomes (KEGG) orthology.

c Fragments per kilobase of exon per million fragments mapped.

The expression levels of *z-iso*, *crtiso* and *al1* genes, which catalysed lycopene biosynthesis, were 4.83, 5.25 and 3.87 times higher in the mutant than in the wild type. Cellular lycopene and carotene increased because the former was the precursor of the latter. The up-regulation of the pigment-relevant genes promoted chlorophyll *a* and carotene synthesis in ZJU9000 and enhanced the efficiency of light utilization.

The pigment measurements showed that on the 4^th^ day, the chlorophyll *a* and carotenoid contents in mutant ZJU9000 increased by 107.1% and 80.6%, respectively (Table 2). The photosynthetic efficiency test results also showed that the *F*_v_/*F*_m_ value of the mutant ZJU9000 was 17.5% higher than the wild type (Table 2). These physiological results affirmed the results of the gene expression analysis on the improvement of pigments and light use efficiency.

**Table 2:** Chlorophyll *a* content, carotenoid content and *F*_v_/*F*_m_ value of *Arthrospira* wild type and mutant on the 4^th^ day

Measurements	Wild type	Mutant ZJU9000
Chlorophyll *a* content (μg ml^−1^)	39.2	81.2
Carotenoid content (μg ml^−1^)	25.0	45.2
*F*_v_/*F*_m_ value	0.4575	0.5375

### 3.3. More energy was provided in ZJU9000

In ZJU9000, growth rate was positively correlated with energy demand during reproductive processes, such as DNA/RNA duplication and protein synthesis.^24^ Correspondingly, the expression levels of genes related to glycolysis, TCA cycle and carbohydrate synthesis in cells were much higher in ZJU9000 than in the wild type, indicating the higher energy supply/demand in the mutant.^25^

#### 3.3.1 Improved carbohydrate synthesis

Starch and sucrose metabolisms were finely co-regulated and were the key steps in the control of carbon flux in plant cells.^26^ The phosphorylation of glucose and fructose, which are the building blocks of complex carbohydrates, were key processes that provided energy to cells.^27^ The carbohydrate metabolism in ZJU9000 cells was more active than that in the wild type. Seven genes in the starch and sucrose metabolic pathways were up-regulated, and no gene was down-regulated (Table 3). The expression levels of sucrose synthase and β-glucosidase genes, which catalyse sucrose and glucose formation (eqn (2) and (3)), respectively, increased by 117% and 138% (Table 1).

2 UDP-glucose + d-fructose → UDP + sucrose

3 d-glucoside + H_2_O → d-glucose + ROH

**Table 3:** Number of regulated genes in key pathways of *Arthrospira* mutant and their biological functions

Pathway	Biological function	Number of up-regulated genes	Number of down-regulated genes	Number of genes both up-regulated and down-regulated	Ratio of up-regulated genes to the DEGs^a^ (%)
Chlorophyll metabolism	Substrate for photosynthesis	6	0	0	100
Pentose phosphate pathway	Provide ribose phosphate and NADPH	9	2	0	82
Purine metabolism	Synthesis of nucleotides: the genetic materials	13	3	1	76
Pyrimidine metabolism	Synthesis of nucleotides: the genetic materials	9	1	2	75
Starch and sucrose metabolism	Key steps in the control of carbon flux	7	0	0	100
Glycolysis	Generate energy (ATP) and NADH	7	2	0	78
TCA cycle	Provide large amount of energy (ATP)	5	1	0	83
Vitamin E biosynthesis	Improve photosynthetic efficiency and promote cell proliferation	12	0	0	100
Vitamin B2 metabolism	Improve photosynthetic efficiency and promote cell proliferation	3	0	1	75
Biotin metabolism	Improve photosynthetic efficiency and promote cell proliferation	4	0	0	100

a DEGs means differentially expressed genes.

Moreover, the expression level of *scrK* gene, which catalysed fructose formation (eqn (4)), was 1.37 times higher in the mutant ZJU9000 than that in the wild type (Table 1).

4 ADP + beta-d-fructose 6-phosphate → ATP + d-fructose

The amount of substrates for the key steps in carbon flux and synthesis in the outer membrane and peptidoglycan layer of *Arthrospira* cells became sufficient when carbohydrate synthesis was improved.^28,29^

#### 3.3.2 Enhanced glycolysis pathway

Glycolysis converted glucose (C_6_H_12_O_6_) into pyruvate (CH_3_COCOO^−^) and H^+^. The free energy released in this process was used for the formation of high-energy molecules adenosine triphosphate (ATP) and nicotinamide adenine dinucleotide (NADH).^30^

**Fig. 4:**
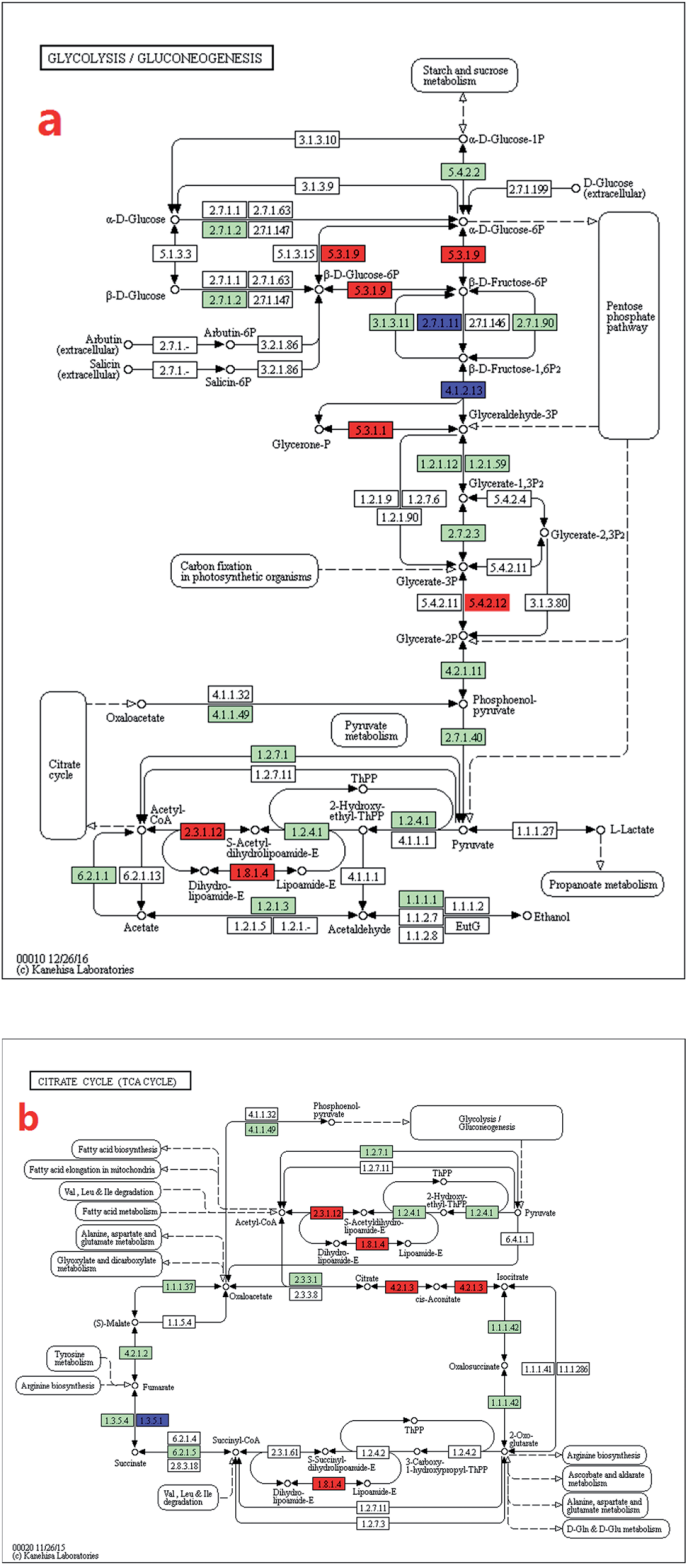
Transcript expression changes involved in energy provision process (glycolysis and TCA cycle) of *Arthrospira* mutant ZJU9000 (coloured boxes are *Arthrospira* specific enzymes on the basis of references, red boxes represent up-regulated genes, blue boxes represent down-regulated genes). (a) Transcript expression changes involved in glycolysis. (b) Transcript expression changes involved in TCA cycle.

The glycolysis-related genes of the mutant cells had higher expression levels than that of the wild type (Fig. 4a). Eight genes were up-regulated, whereas two genes were down-regulated (Table 3). The expression level of *gpi* gene, which catalysed the key step of glucose conversion into fructose (eqn (5)), increased by 232% (Table 1) and enhanced the glycolytic reactions, thereby providing more energy for the cells.

5 α/β d-glucose 6-phosphate → beta-d-fructose 6-phosphate

The expression level of *dld* gene increased by 216%, and this increase promoted H^+^ synthesis (eqn (6)).

6 enzyme N6-(dihydrolipoyl)lysine + NAD^+^ → enzyme N6-(lipoyl)lysine + NADH + H^+^

#### 3.3.3 Enhanced TCA cycle pathway

TCA cycle released stored energy into large amounts of ATP and CO_2_ through the oxidation of acetyl-CoA derived from carbohydrates, fats and proteins.^31^ When the energy supply was inadequate (high ADP level and low ATP and NADH levels), the TCA cycle was accelerated such the cells received sufficient ATP and NADH.^32^

The expression levels of TCA cycle genes were higher in mutant cells than in wild type cells (Fig. 4b). The expressions of *dlat* and *dld* genes, which jointly catalysed pyruvate into acetyl-CoA (the substrate of TCA cycle), increased by 113% and 216%, respectively (Table 1). In the second step of the TCA cycle, citrate was turned into isocitrate under aconitase hydratase (*aco*) catalysis (eqn (7)). As the expression level of *aco* gene increased by 162%, second step of the TCA cycle was enhanced and TCA cycle was driven cycling to generate more substrate for ATP synthesis, providing more energy for cell activities.

7 Citrate → isocitrate

### 3.4. The amount of synthesized genetic materials for cell proliferation increased in ZJU9000

The biomass yield of ZJU9000 was 176% higher than the wild type on the 4^th^ day, and the rate of cell proliferation in the former increased, thereby increasing the amount of substrates of genetic materials, such as nucleotides and ribose 5-phosphate. This improvement was verified by the increased expression levels of genes related to ribose 5-phosphate, purine and pyrimidine nucleotides biosynthesis.

#### 3.4.1 Enhanced pentose phosphate pathway

**Fig. 5:**
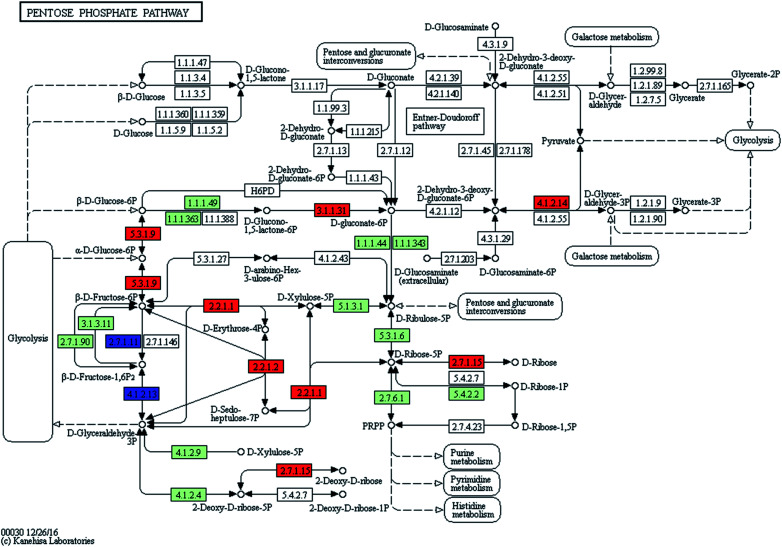
Transcript expression changes involved in pentose phosphate pathway of ZJU9000 (coloured boxes are *Arthrospira*-specific enzymes on the basis of references, red boxes represent up-regulated genes and blue boxes represent down-regulated genes).

The pentose phosphate pathway was a glucose turnover process that produced NADPH as a reducing equivalent and pentose as an essential part of nucleotides.^33^ A notable up-regulated expression of ribose 5-phosphate-related genes was observed in the pentose phosphate pathway of ZJU9000 (Fig. 5). The expression of *gpi*, *tkt* and *tal* genes, which catalysed the reaction cascade ‘glucose 6-phosphate → fructose 6-phosphate → ribose 5-phosphate’, increased by 232%, 121% and 345%, respectively (Table 1). The expression of *rbks* gene, which catalysed eqn (8) to generate more d-ribose 5-phosphate, was 2.91 times higher in the mutant.

8 ATP + d-ribose → ADP + d-ribose 5-phosphate

The increased ribose 5-phosphate levels in ZJU9000 facilitated the formation of phosphoribosyl pyrophosphate, which is an activated compound used in the biosynthesis of histidine and purine or pyrimidine nucleotides.

#### 3.4.2 Improved purine and pyrimidine nucleotide biosynthesis

Purine and pyrimidine nucleotides were fundamental to life given their involvement in nearly all biochemical processes. Purine and pyrimidine nucleotides were the monomeric units of DNA and RNA.^34^ In the mutant ZJU9000, in terms of purine and pyrimidine metabolism, 22 genes were up-regulated, and 4 were down-regulated (Table 3). The expression levels of *gmk* and *itpa* genes, which catalysed guanylic acid (GMP) formation, increased by 313% and 406%, respectively (Table 1). The levels of the *umps* and *itpa* genes, which catalysed uridylic acid formation, increased by 1.61 and 4.06 times, respectively (Table 1).

The amount of synthesized genetic materials increased in individual mutant cells after ^60^Co gamma-ray irradiation, and these genetic materials provided the material foundation for rapid cell proliferation.

### 3.5. More vitamins were synthesized in ZJU9000

**Fig. 6:**
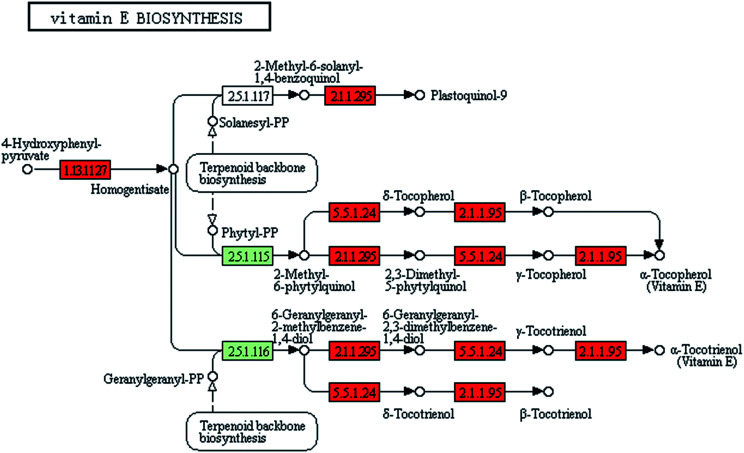
Transcript expression changes involved in vitamin E biosynthesis of ZJU9000 (coloured boxes are *Arthrospira*-specific enzymes on the basis of references and red boxes represent up-regulated genes).

The gamma-ray irradiation extensively induced the overexpression of vitamin synthesis-related genes in ZJU9000. Of the 14 known genes related to vitamin E synthesis, 12 were up regulated, such as *vte1*, *vte3* and *vte4* (Fig. 6), which eventually exhibited enhanced light use efficiency and reproductive performance.^35–37^ The expression levels of *ribD*, *bioF* and *fabG* genes, which catalysed vitamin B2 and biotin biosynthesis, increased by 320%, 1131% and 164%, respectively (Table 1). These improvements promoted vitamin B2 and biotin formation and enhanced the final cell density.^38^

Phylloquinone (vitamin K1) was a ubiquitous photosynthetic plant compound and functioned as a photosystem I-mediated electron transport cofactor. Menaquinone (vitamin K2) was an obligatory electron-transfer pathway component in bacteria.^39^ In ZJU9000, the expression level of the *ubiE* gene, which catalyses vitamin K1 and K2 synthesis (eqn (9) and (10)), increased by 107%. Thus, the mutant had increased the amount of synthesized vitamin K1 and K2 and exhibited an improved electron transport in its photosynthetic processes.

9 2-Phytyl-1,4-naphthoquinone + S-adenosyl-l-methionine → vitamin K1 + S-adenosyl-l-homocysteine

10 2-Demethylmenaquinone + S-adenosyl-l-methionine → vitamin K2 + S-adenosyl-l-homocysteine

Vitamins were important in the cellular activities of microalgae. Transcriptome sequencing proved that in our study, ^60^Co gamma-ray irradiation enhanced the synthesis of numerous vitamins in mutant cells, improved the photosynthetic efficiency and promoted cell proliferation.

### 3.6. Enhancing carbon concentration mechanism (CCM) in ZJU9000

Low CO_2_ concentrations impeded photosynthesis in most plants,^40^ and carbon concentration mechanism was vital in photosynthesis and facilitated carbon assimilation *via* the Calvin cycle.^41^

As the experiment conditions were air bubbling (with 0.038 vol% CO_2_), CCM in the C4 pathway was activated. The carbon utilization efficiency of *Arthrospira* depended on the capacity of CCM. The expression of *maeB* gene, which catalysed the release of concentrated CO_2_ for cell usage (eqn (11)), increased by 120% in the carbon fixation pathway of ZJU9000 (Table 1). Gamma-ray irradiation enhanced CCM in ZJU9000, thus efficiently providing concentrated CO_2_ for cells and promoting CO_2_ biofixation.

11 Malate + NAD(P)^+^ → pyruvate + CO_2_ + NAD(P)H + H^+^

## 4. Conclusions

High-throughput sequencing was used for the systematic analysis of pathway expression levels in wild type *Arthrospira* and ZJU9000. The purpose was to elucidate the improved growth phenotype of the mutant. The enhanced synthesis of key pigments and vitamins in ZJU9000 improved the photosynthesis of cells and accelerated cell growth. The increase in the amount of generated nucleotides and ATP provided genetic material and an energetic basis for rapid cell proliferation. The enhancement of CCM improved the utilization of CO_2_ at a low concentration (0.038 vol% in air). All factors jointly contributed to the robust growth of ZJU9000.

## Conflicts of interest

There are no conflicts to declare.

## Supplementary Material

RA-008-C8RA01626G-s001
