# Supplementary material for: Enhancing growth-relevant metabolic pathways of Arthrospira platensis (CYA-1) with gamma irradiation from 60Co
Source: RSC Adv. 2018 May 8;8(30):16824–33. doi: 10.1039/c8ra01626g (PMC9080328; doi:10.1039/c8ra01626g)
Supplement: RA-008-C8RA01626G-s001 [file RA-008-C8RA01626G-s001.pdf]

Electronic Supplementary Material (ESI) for RSC Advances.
This journal is © The Royal Society of Chemistry 2018

Specific procedures for RNA extraction, RNA library preparation and bioinformatics analysis

Total RNA was extracted using the mirVana miRNA Isolation Kit (Ambion) according to manufacturer protocols. RNA integrity was evaluated using the Agilent 2100 Bioanalyzer (Agilent Technologies, Santa Clara, CA, USA). Samples with RNA Integrity Number (RIN) > 7 were subjected to subsequent analysis. The libraries were constructed using TruSeq Stranded mRNA LTSample Prep Kit (Illumina, San Diego, CA, USA) according to the manufacturer instructions. These libraries were then sequenced on the Illumina sequencing platform (HiSeqTM 2500) and 150 bp/125bp paired-end reads were generated.

1 RNA extraction

(A) A volume of 600 μL Lysis/Binding Buffer was added to the cell followed by addition of 30 μL miRNA homogenate additive and vortex mixing. The mixture was then left on the ice bath for 10 min.

(B) Phenol:chloroform was added at a volume equal to lysate volume before adding the miRNA Homogenate Additive. Samples were centrifuged for 5 min at maximum speed (10,000 x g) to separate the aqueous and organic phases, removing and transferring the former to a fresh tube.

(C) Ethanol was added to the aqueous phase at 1.25 times of the latter volume.

(D) The lysate/ethanol mixture was loaded onto the filter cartridge and centrifuge for 30 s at 13000 rpm, discarding the flow-through.

(E) A 350 μL volume of miRNA wash solution 1 was added to the filter cartridge and centrifuged for 30 s at 13000 rpm, discarding the flow-through.

(F) A mixture of 10 μL DNase I and 70 μL Buffer RDD QIAGEN (#79254) was added to the filter cartridge before leaving the cartridge at room temperature for 15 min.

(G) 350 μL volume of miRNA wash solution 1 was added to the filter cartridge and centrifuged for 30 s at 13000 rpm, discarding the flow-through.

(H) 500 μL volume of wash solution 2/3 was added to the filter cartridge and centrifuged for 30 s at 13000 rpm twice, discarding the flow-through

(I) The assembly was spun for 1 min to remove residual fluid from the filter. A 100-μL volume of pre-heated (95 °C) elution solution was loaded on the filter before left at room temperature for 2 min. Samples were spun for 20–30 s at maximum speed to recover the RNA eluate before storing at –70 °C.

2 RNA library preparation

(A) Purify and Fragment mRNA

Each well containing 50 μL total RNA (4 μg) was added and thoroughly mixed with 50 μL *RNA purification beads*. The mixture was incubated at 65 °C for 5 min, removed at 4 °C and placed at room temperature for 5 min. The supernatant was discarded from each well. A 200 μL volume of bead washing buffer was added and mixed thoroughly in each well. The mixture was placed on the magnetic stand at room temperature for 5 min before the supernatant was discarded from each well. A

50 μL elution buffer was added and mixed thoroughly in each well. The samples were incubated at 80 °C for 2 min before placing at room temperature. A 50-μL beads binding buffer was added in each well and mixed thoroughly at room temperature for 5 min; the supernatant was discarded from each well. Bead washing buffer at 200 μL was in each well and mixed thoroughly at room temperature for 5 min. The supernatant was discarded from each well. A 19.5 μL of *Elute, Primer, Fragment Mix* was added in each well and mixed thoroughly. The mixture was incubated at 94 °C for 8 min and removed at 4 °C before centrifugation.

(B) Synthesise First Strand cDNA

a. 8 μL of *First Strand Synthesis Act D Mix* and *SuperScript II Reverse Transcriptase* was added in each well and mixed thoroughly. The mixture was incubated at 25 °C for 10 min, 42 °C for 15 min and 70 °C for 15 min and held at 4 °C afterwards.

(C) Synthesise Second Strand cDNA

Each well was added with 5 μL diluted *End Repair Control* (2 μL *End Repair Control* + 98 μL *Resuspension Buffer*) and 20 μL *Second Strand Marking Master Mix* and mixed thoroughly. The mixture was incubated at 16 °C for 60 min before adding and thoroughly mixing with 90 μL mixed *AMPure XP beads* at room temperature for 15 min. The mixture was placed on a magnetic stand at room temperature for 5 min. The supernatant was discarded each well. A 200 μL of 80% ethyl alcohol was added to each well and mixed for 30 s; the supernatant was discarded each well, and this step was repeated twice. The mixtures were air-dried for 15 min at room temperature.

A 17.5 μL of resuspension buffer was added and mixed thoroughly before incubation at room temperature for 2 min. Mixtures were placed on the magnetic stand at room temperature for 5 min to allow transfer of 15 μL supernatant from each well into the new PCR plate.

(D) Adenylate 3'Ends

Each well was added and thoroughly mixed with 2.5 μL diluted *A-Tailing Control* (1 μL *A-Tailing Control* + 99 μL *Resuspension Buffer*) and 12.5 μL *A-Tailing Mix*. The mixture was incubated at 37 °C and 70 °C for 30 min and 5 min, respectively, before holding at 4 °C.

(E) Ligate Adapters

Each well was added and mixed thoroughly with 2.5 μL diluted *Ligation Control* (1 μL Ligation Control + 99 μL *Resuspension Buffer*), 2.5 μL *Ligation Mix* and 2.5 μL *RNA Adapter Index* at 30 °C for 10 min. A 5 μL *Stop Ligation Buffer* was added and thoroughly mixed in each well and mix thoroughly before adding and mixing in 42 μL of mixed *AMPure XP beads* at room temperature for 15 min, discarding 79.5 μL of the supernatant from each well. 200 μL of 80% ethyl alcohol was added to each well and placed at room temperature for 30 s, after which the supernatant was discarded, and the same step was repeated. The mixtures were air-dried for 15 min at room temperature. 52.5 μL *Resuspension Buffer* was added and mixed thoroughly before incubating at room temperature for 2 min. The mixtures were then placed on the magnetic stand at room temperature for 5 min, transferring 50 μL of the supernatant from each well into the new PCR plate. The wells of the new PCR plate were added

and thoroughly mixed with 50 μL mixed *AMPure XP beads* for 15 min. The plate was placed on the magnetic stand at room temperature for 15 min, discarding the 95 μL of the supernatant from each well afterwards. 200 μL of 80% ethyl alcohol was added to each well and placed on a magnetic stand at room temperature for 30 s, discarding the supernatant afterwards and repeating the same step. The mixtures were air-dried for 15 min at room temperature. 22.5 μL *Resuspension Buffer* was added and mixed thoroughly in each well at room temperature for 2 min. The mixtures were then placed on the magnetic stand at room temperature for 5 min, transferring 20 μL of the supernatant from each well into another PCR plate.

(F) Enrich DNA Fragments

Each well was added and thoroughly mixed with 5 μL PCR Primer Cocktail and 25 μL PCR Master Mix. The mixture was incubated in 1 cycle of 98 °C for 30 s, 15 cycles of 98 °C for 10 s, 60 °C for 30 s and 72 °C for 30 s and 1 cycle of 72 °C for 5 min, before holding at 10 °C.

(G) Purification

Each well was added and thoroughly mixed with 50 μL mixed *AMPure XP beads*, at room temperature for 15 min. The mixtures were placed on a magnetic stand at room temperature for 15 min, discarding 95 μL of the supernatant from each well. 200 μL of 80% ethyl alcohol was added to each well and placed on a magnetic stand at room temperature for 30 s, discarding the supernatant afterwards and repeating the same step. The mixtures were air-dried for 15 min at room temperature. 32.5 μL of *Resuspension Buffer* was added and mixed thoroughly in each well. The mixture was

incubated at room temperature for 2 min. The mixtures were placed on the magnetic stand at room temperature for 5 min before transferring 30 μL of the supernatant from each well into the new PCR plate.

(H) Validate library

A 1 μL volume of the sample was loaded on an Agilent 2100 Bioanalyzer to determine library size and purity.

3 Bioinformatics analysis

(A) Quality control and mapping

Raw data were processed using NGS QC Toolkit [1]. The reads containing ploy-N and the low quality reads were removed to obtain clean reads. The clean reads were then mapped to *Arthrospira sp.* PCC 8005 (accession number “NZ_FO818640”) using Tophat [2].

(B) Analysis of differentially expressed genes (DEGs), cluster analysis, GO and KEGG enrichment

Fragments Per Kilobase Of Exon Per Million Fragments Mapped (FPKM) value of each gene was calculated using cufflinks [3], and the read counts of each gene were obtained by htseq-count [4]. DEGs were identified using the DESeq (2012) functions, estimateSizeFactors and nbinomTest [5]. A P value < 0.05 was set as the threshold for significantly differential expression. Hierarchical cluster analysis of DEGs was performed to explore the gene expression pattern. Gene ontology (GO) and KEGG pathway enrichment analyses of DEGs were performed using the correlation coefficient based on the hypergeometric distribution.

References

1 R. K. Patel and M. Jain, *PLoS One*, , DOI:10.1371/journal.pone.0030619.

2 D. Kim, G. Pertea, C. Trapnell, H. Pimentel, R. Kelley and S. L. Salzberg, *Genome Biol*, 2013, **14**, R36.

3 C. Trapnell, A. Roberts, L. Goff, G. Pertea, D. Kim, D. R. Kelley, H. Pimentel, S. L. Salzberg, J. L. Rinn and L. Pachter, *Nat Protoc*, 2012, **7**, 562–78.

4 S. Anders, P. T. Pyl and W. Huber, *Bioinformatics*, 2015, **31**, 166–169.

5 S. Anders and W. Huber, *Eur Mol Biol Lab*.
